# Supplementary figures and images for: IL18 Receptor Signaling Inhibits Intratumoral CD8+ T-Cell Migration in a Murine Pancreatic Cancer Model
Source: Cells. 2023 Jan 31;12(3):456. doi: 10.3390/cells12030456 (PMC9913970; doi:10.3390/cells12030456)

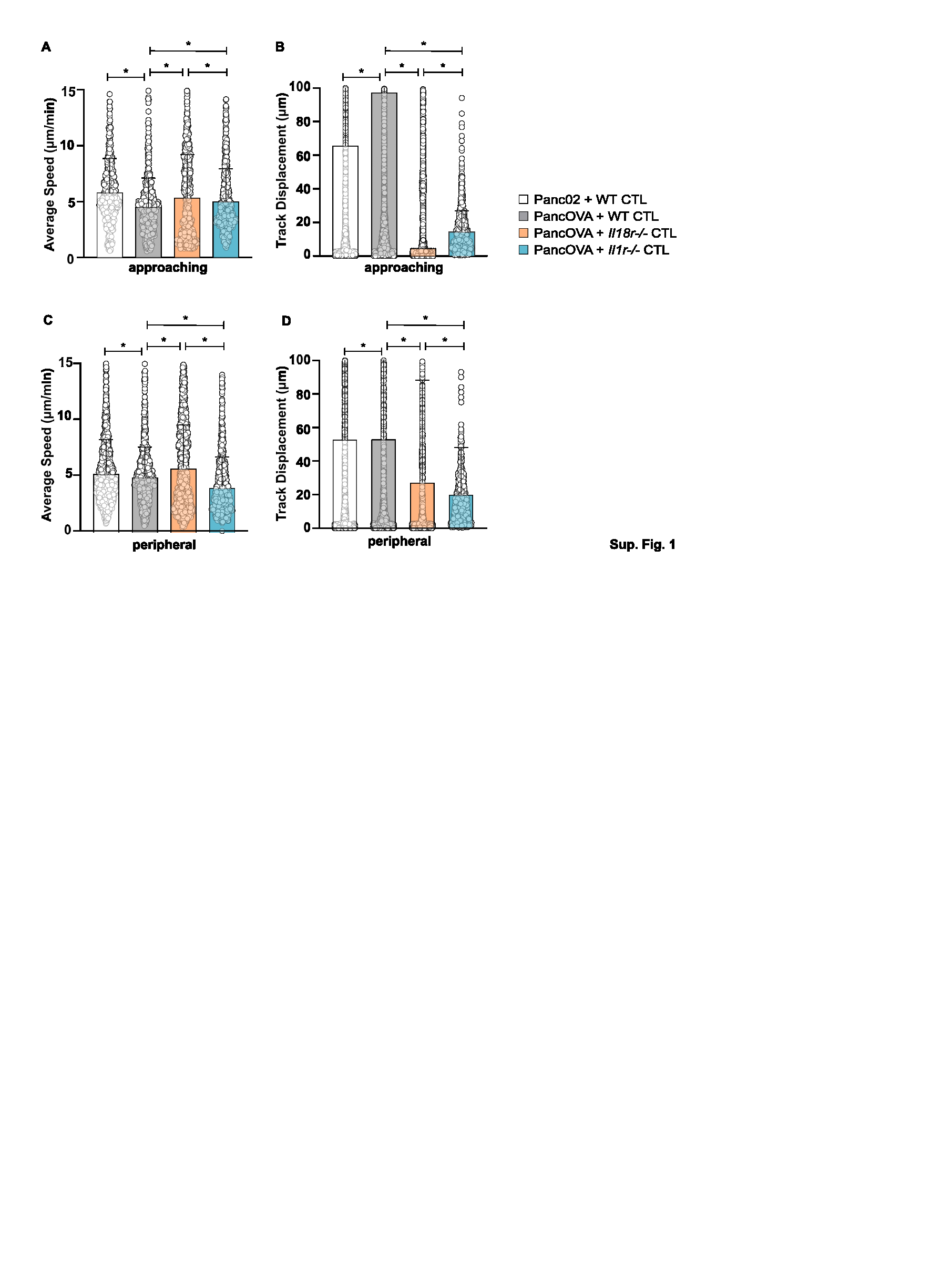

Supplement: Supplementary file 1 [file cells-12-00456-s001.zip › Sup 1_221207.tiff]

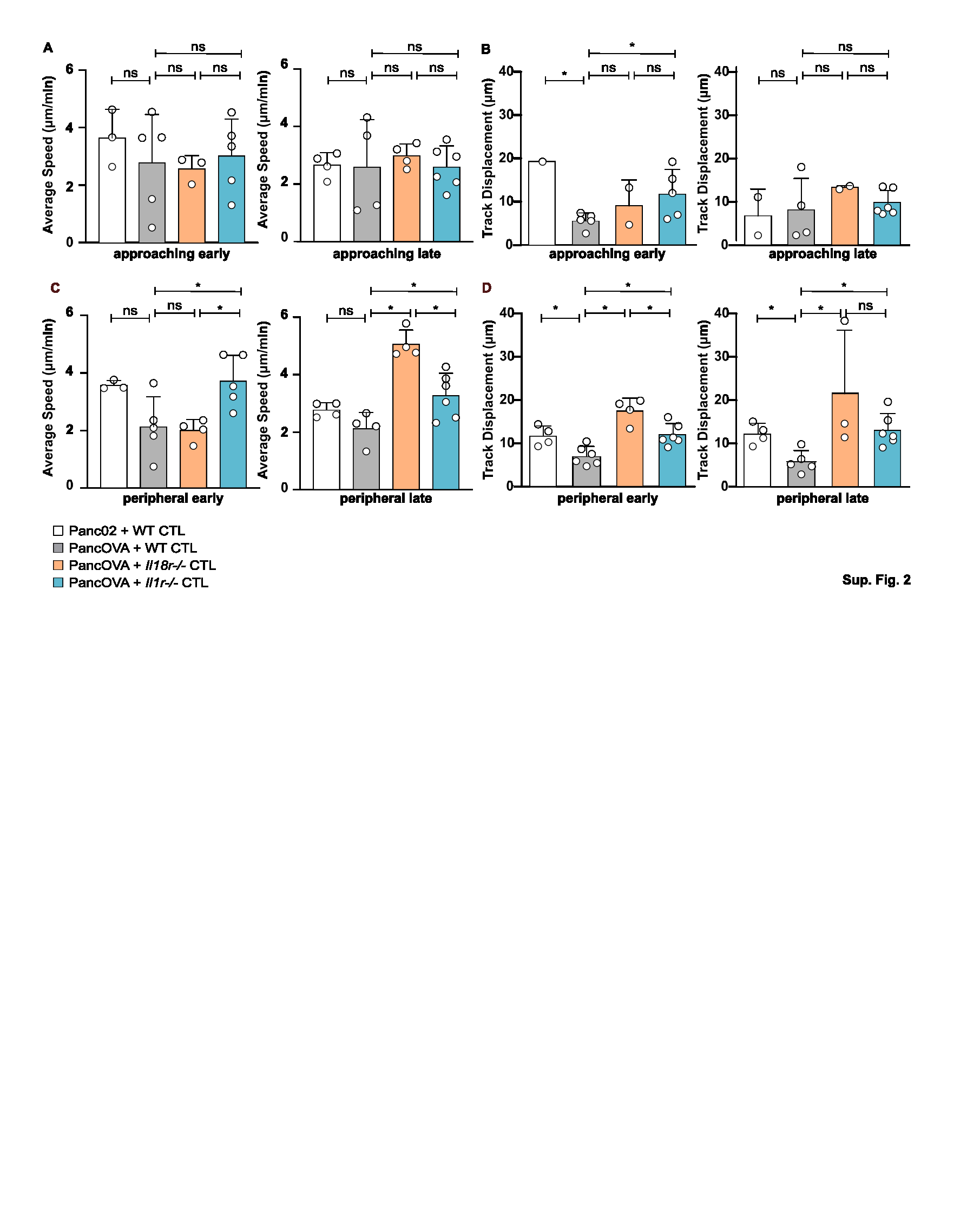

Supplement: Supplementary file 1 [file cells-12-00456-s001.zip › Sup 2_221204.tiff]
